# Supplementary material for: Implications of Metastable Nicks and Nicked Holliday Junctions in Processing Joint Molecules in Mitosis and Meiosis
Source: Genes (Basel). 2020 Dec 12;11(12):1498. doi: 10.3390/genes11121498 (PMC7763299; doi:10.3390/genes11121498)
Supplement: Supplementary file 1 [file genes-11-01498-s001.pdf]

Implications of metastable nicks and nicked Holliday junctions in processing joint molecules in mitosis and meiosis

Félix Machín

Unidad de Investigación. Hospital Universitario Nuestra Señora de Candelaria. Santa Cruz de Tenerife, Spain.
Instituto de Tecnologías Biomédicas, Universidad de la Laguna, Tenerife, Spain.
Facultad de Ciencias de la Salud, Universidad Fernando Pessoa Canarias, Las Palmas de Gran Canaria, Spain.
* Correspondence: fmachin@fciisc.es

SUPPLEMENTARY FIGURES

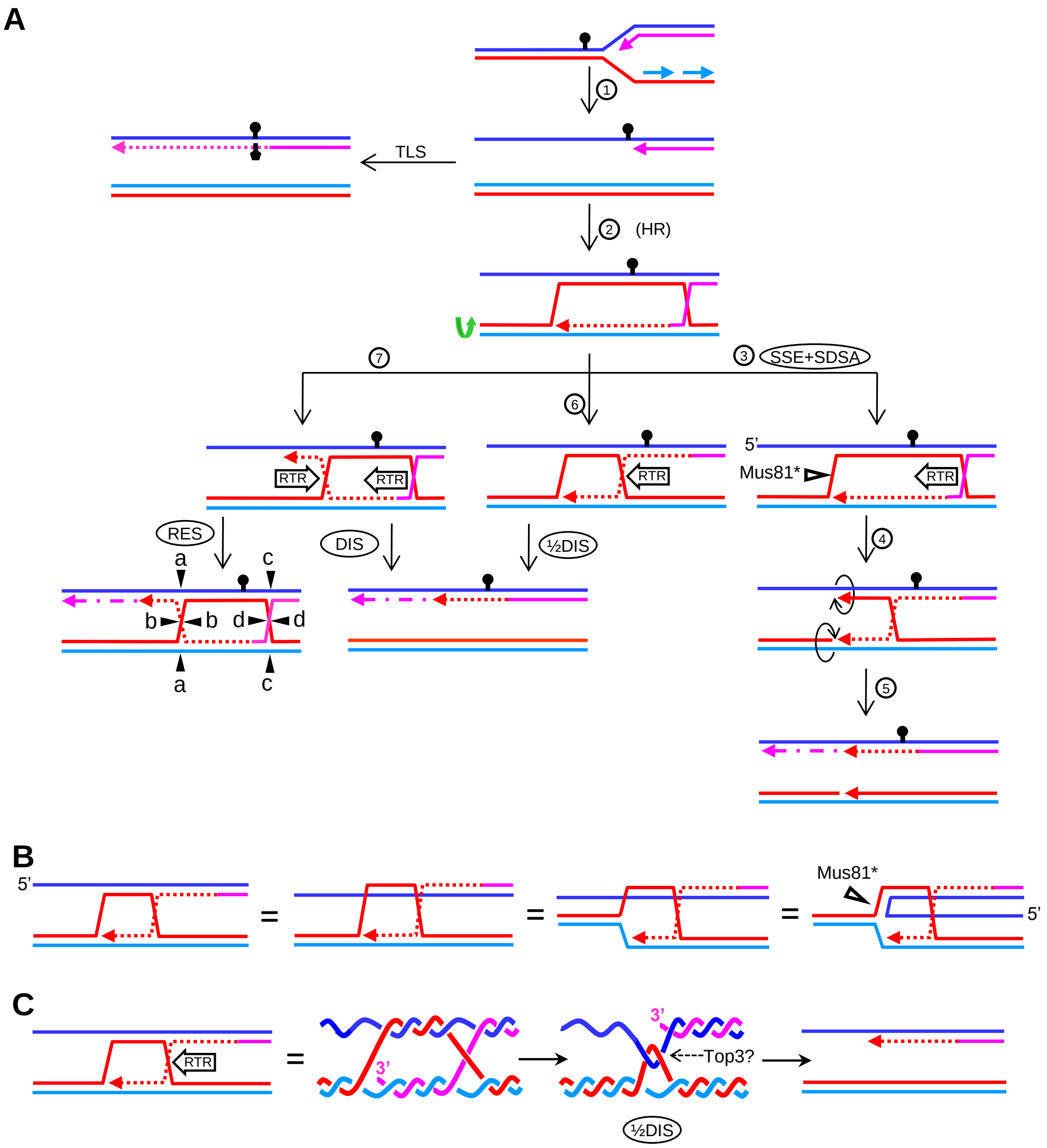

Figure S1. Bypass of replication blockage at the leading strand. (**A**) General model: (1) A DNA adduct (black bulb) blocks synthesis of the leading strand during replication, leaving a ssDNA tract as the RF walks ahead; (2) the 3′ end melts and invades the sister chromatid, which acts as a donor to prime DNA synthesis and bypass the blockage, while the displaced strand is captured by the ssDNA tract, resulting in a JM that comprises a cHJ and a pseudo-HJ with three dsDNA arms a fourth arm comprising ssDNA; (3-5) the JM is processed by first resolving the pseudo-HJ with an SSE (Mus81* appears suitable for this task), followed by branch migration of the cHJ towards the 3′ end and its opposing nick (RES+SDSA-like); (6) alternatively, the cHJ could migrate towards the pseudo-HJ and both are eliminated through a half dissolution pathway (½DIS); (7) the pseudo-HJ branch migrates to become a cHJ, resulting in a DPE dcHJ, which can then be processed as in Figure 7. The alternative non-HR translesion synthesis pathway (TLS) is also depicted. (**B**) A visual conversion of the pseudo-HJ into the equivalent RF Y-like structure with a regressed leading strand, a known substrate of Mus81*. As depicted, Mus81* cuts in the crossover strand (red) in the pseudo-HJ. (**C**) A detailed look at the half dissolution pathway that could deal with the cHJ/pseudoHJ partner. Blue line (Watson strand) and red line (Crick strand) make the parental dsDNA. Pink and cyan lines depict the newly-synthetized strands during replication. Red dotted lines mark HR-driven DNA synthesis, whereas a dash-dotted pink arrows mark post-HR synthesis. The arrowhead indicates the 3′ end. Green arched arrow shows a visual vertical flipping of a sister chromatid.

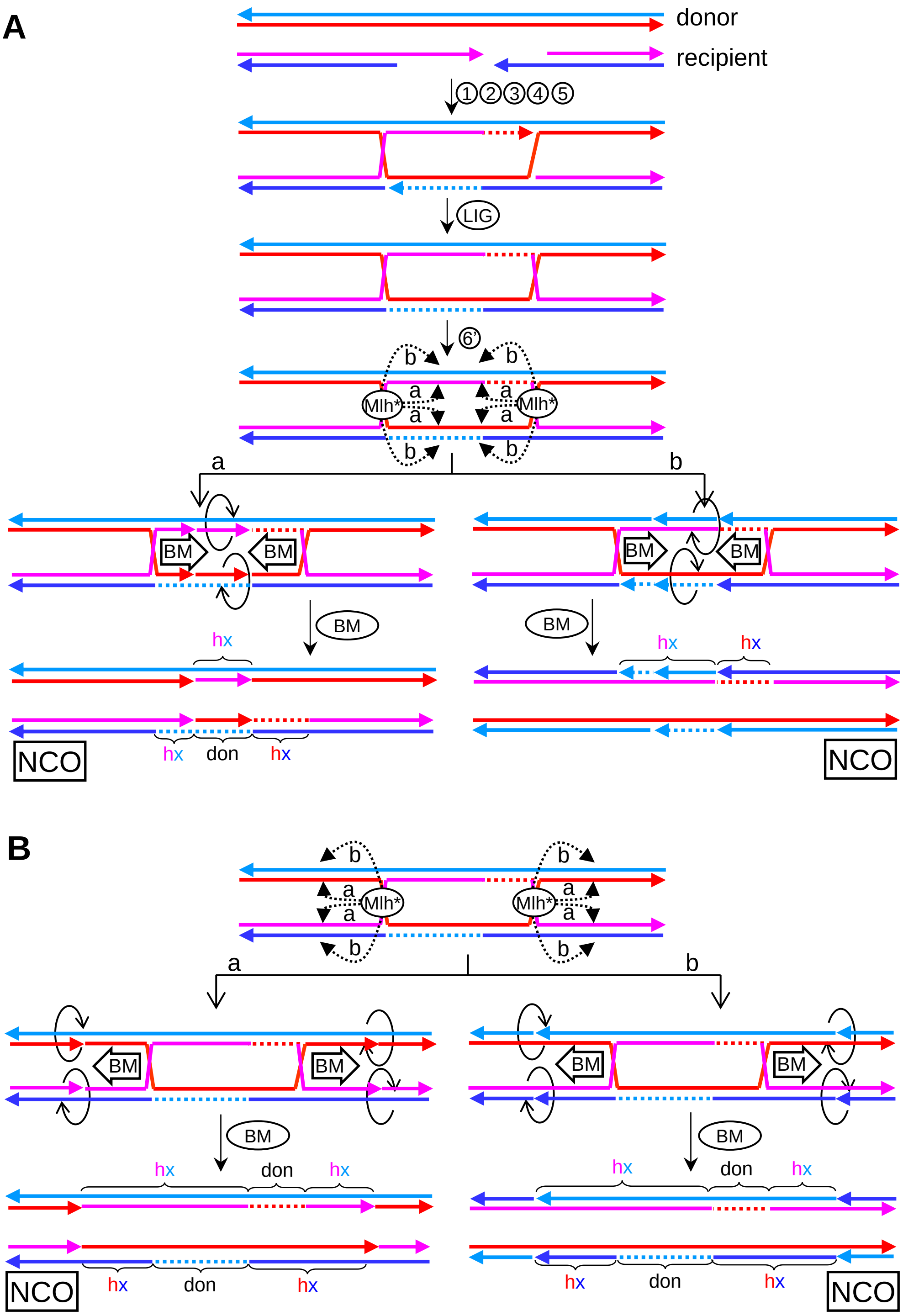

Figure S2. Mlh1-Mlh3 ZMM meiotic resolution upon DPE dHJs results in NCOs when crossover ("a") or noncrossover ("b") strand specificity applies in the model with incisions in *trans*. Steps (1-5) and (LIG) like in Figure 7A. To make the incisions (step 6′), Mlh1-Mlh3 could polymerize inwards (**A**) or outwards (**B**). Then, branch migration towards the opposing nicks results in the resolution of the cHJs. NCO, non-crossover; LIG, ligation; BM, branch migration; Mlh*, Mlh1-Mlh3 heterodimer; don, donor sequence tracts; hx, heteroduplex DNA tracts (the color of each letter encodes the strands involved in the heteroduplex). Blueish lines, Crick strands; reddish lines, Watson strands. Dark and light colors are also included so that the four strands can be differentiated: Red and cyan are complementary donor strands, whereas pink and blue are complementary recipient strands. The arrowhead indicates the 3′ end. Colored dotted lines mark HR-driven newly synthesized sections.

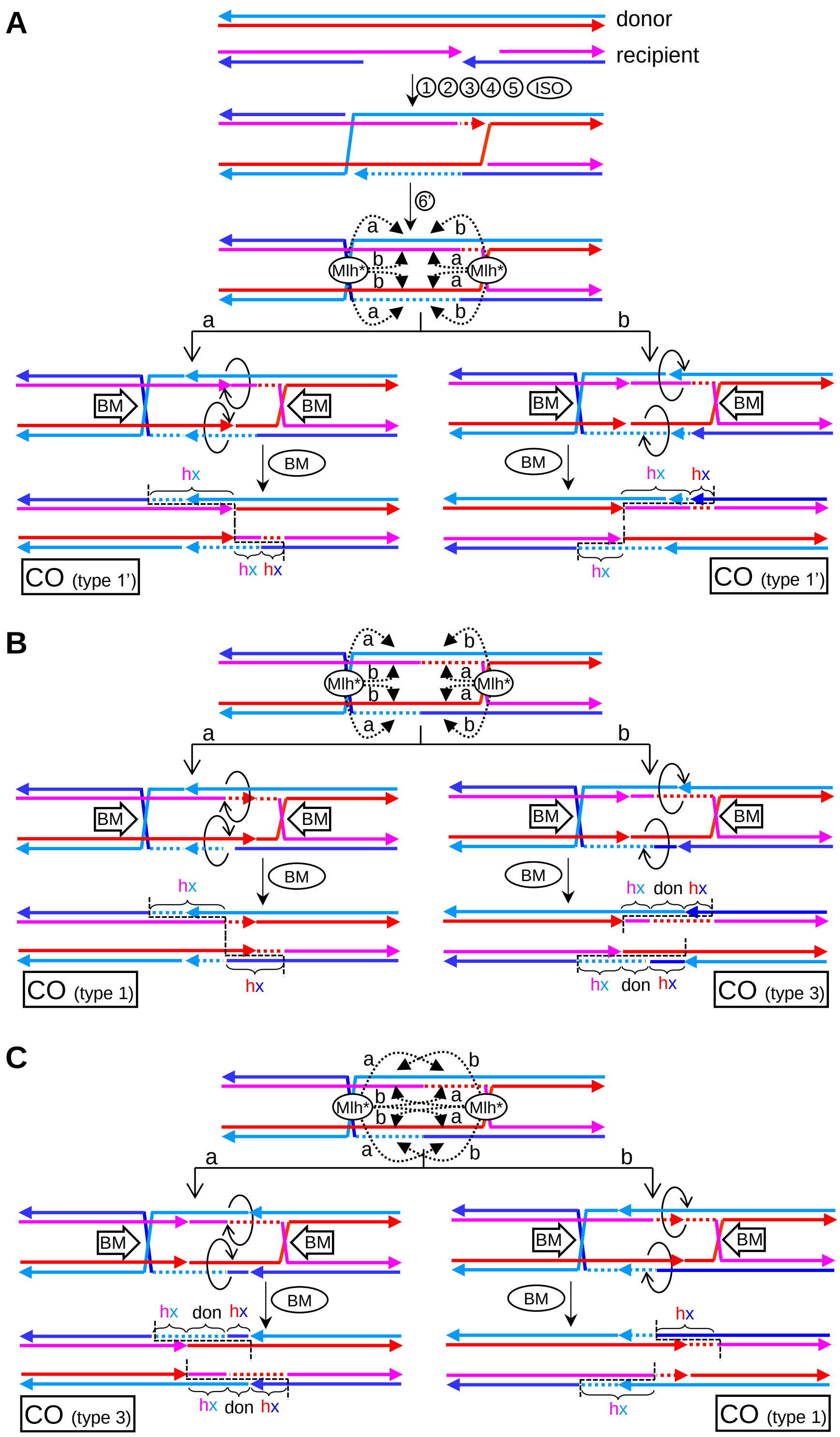

Figure S3. Variations of the inward nicking scenario shown in Figure 10A. (**A**) Only one incision takes place in a newly-synthesized section. Steps (1-5) and ISO are like in Figure 7. The length of the HR-driven synthesis is much larger after the second end capture than during the first invading event, so that this alternative Figure 10A scenario can be accomplished. In this scenario, type 1′ COs are the outcomes irrespective of the crossover strand specificity ("a" or "b"). Type 1′ CO is a type 1 CO with a strand swap in the middle of the hxDNA of one of the genetic products. (**B**) Incisions affect both newly-synthesized sections in the crossover strand specificity ("a") and none in the non-crossover strand specificity ("b"). Type 1 CO is the outcome for "a". (**C**) Incisions affect both two newly-synthesized tracts in the non-crossover strand specificity ("b"). Type 1 CO is the outcome in this case as well. See previous figure legends for abbreviations and signs.

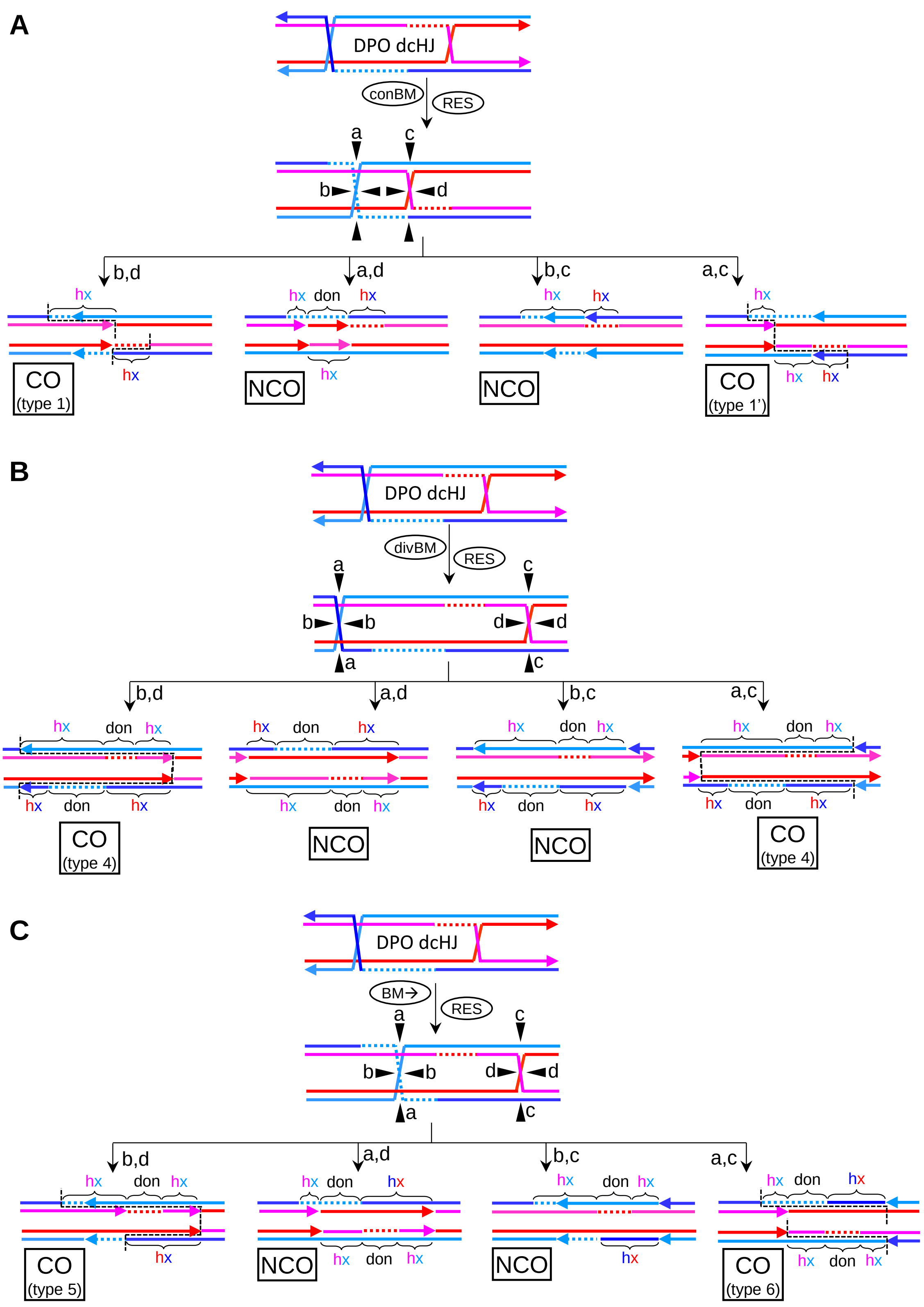

Figure S4. Equivalent CO products to those observed in Mlh1-Mlh3 incisions in *trans* can be obtained in DSBR by coupling branch migration and SSE resolution. (**A**) Inward branch migration precedes resolution to obtain CO products like those shown in Figure 10A by Mlh1-Mlh3 resolution. (**B**) Outward branch migration precedes resolution to obtain CO products like those shown in Figure S6A (below). (**C**) Co-directional branch migration precedes resolution to obtain CO products like those shown in Figure S6B (below). Unlike Mlh1-Mlh3 resolution, NCO products are also expected for SSE resolution. See previous figure legends and Figure 7 for abbreviations and signs.

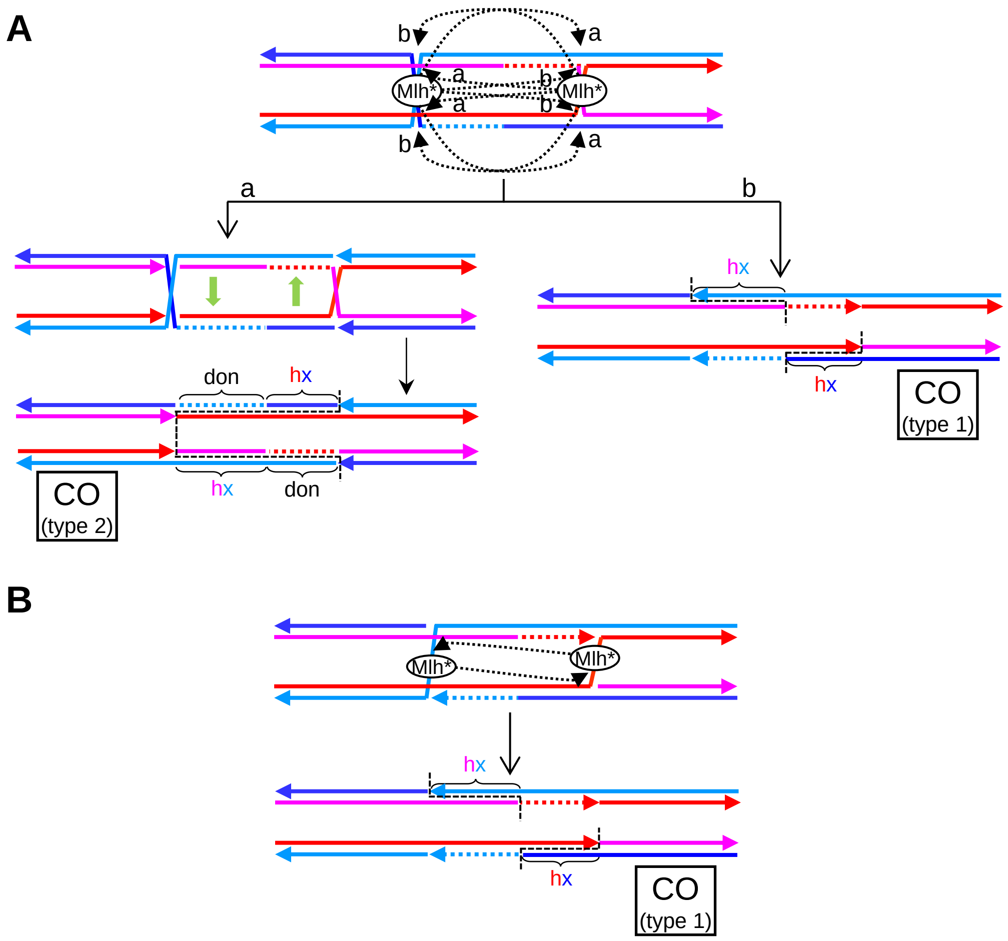

Figure S5. Variation of the inward nicking scenario shown in Figure 10A, whereby Mlh1-Mlh3 directly resolves a HJ in *trans*. (**A**) The substrate is a DPO dcHJ. Each cHJ nucleates a Mlh1-Mlh3 polymer, which then searches and double-nicks the other cHJ. Only the non-crossover strand specificity relative to the nucleating HJ ("b") guarantees type 1 CO through cutting in the crossover strands of the second cHJ (DPO isomer is needed). (**B**) The substrate is a DPO dnHJ. In this case, only type 1 COs are the expected products as cutting in *trans* must take place upon the crossover strand. Green arrows show that the next step is a simple visual relocation of the indicated tracts. See previous figure legends and Figure 10 for other abbreviations and signs.

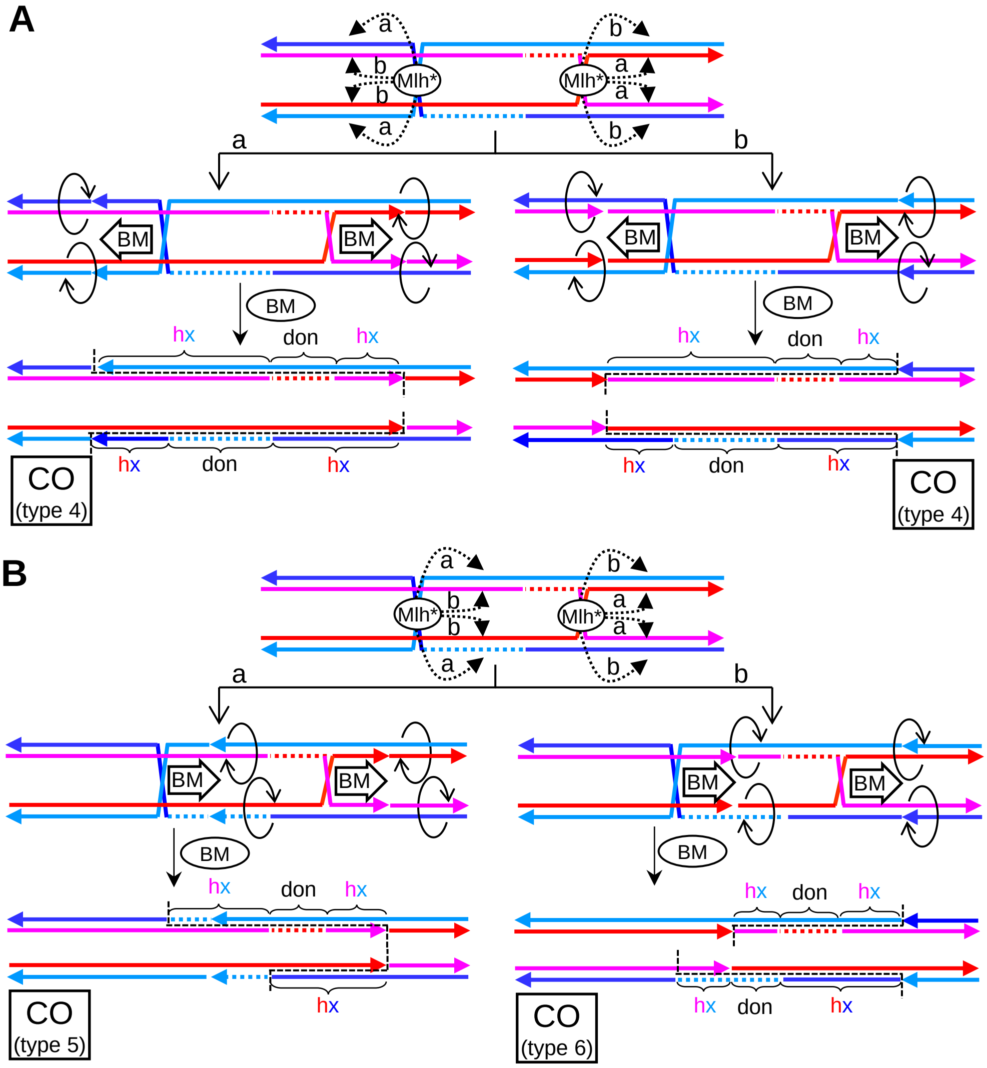

Figure S6. Models of ZMM meiotic resolution based on Mlh1-Mlh3 making non-inward incisions in *trans*. The DPO dHJ is formed as depicted in in Figure 7 and 10A. (A) Mlh1-Mlh3 polymerizes outwards from the cHJs/nHJs (cHJs are depicted in this example). The main restriction of the model is that incisions by Mlh1-Mlh3 take place with strand specificity: "a" for crossover strands and "b" for non-crossover strands. Then, branch migration towards the opposing nicks results in the resolution of the cHJs. Type 4 (inverted specular bidirectional hx-donor-hx tracts with distinct hx strand composition) COs are expected. (**B**) Co-directional incisions by Mlh1-Mlh3 would yield more complex COs (type 5 and 6; which, in turn, are different combinations of types 1 to 4). See previous figure legends for other abbreviations and signs.

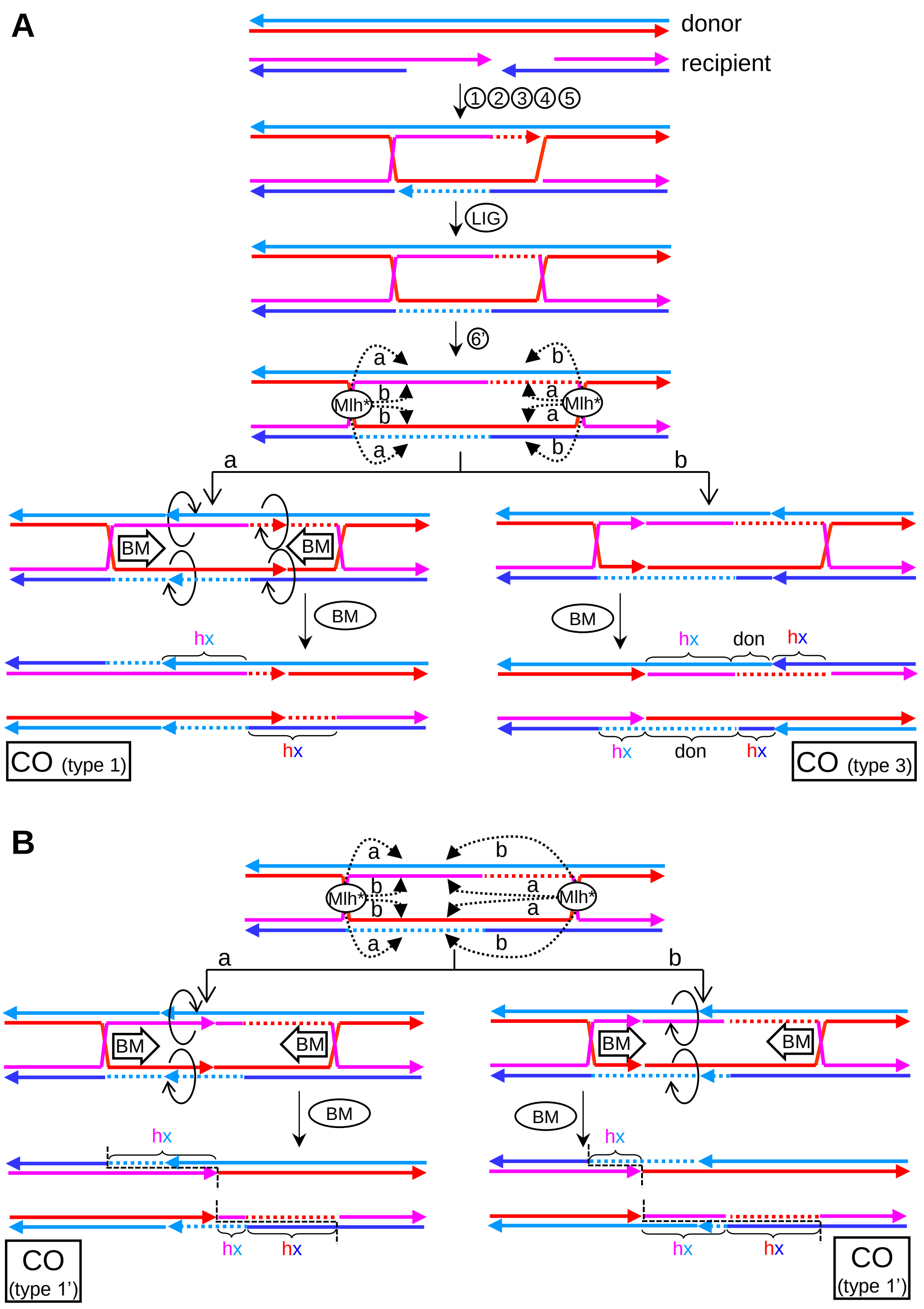

Figure S7. Variations of the inward nicking scenario shown in Figure 10B (DPE dcHJs). (**A**) Incisions affect both newly-synthesized sections in the "a" specificity and none in the "b" specificity. The specificity is given by recognizing newly-synthesized strand sections and then nicking in that section ("a") or its complementary strand ("b"). Type 1 CO is the outcome for "a". (**B**) Only one incision takes place in a newly-synthesized section. The same specificity restriction applies, but one Mlh1-Mlh3 polymer surpasses the DSB site before making the incisions. In this scenario, type 1' COs are the outcomes irrespective of the strand specificity ("a" or "b"). Type 1' CO is a type 1 CO with a strand swap in the middle of the hxDNA of one of the genetic products. Steps (1-5) and LIG are like in Figure 7A. See previous figure legends for abbreviations and signs.

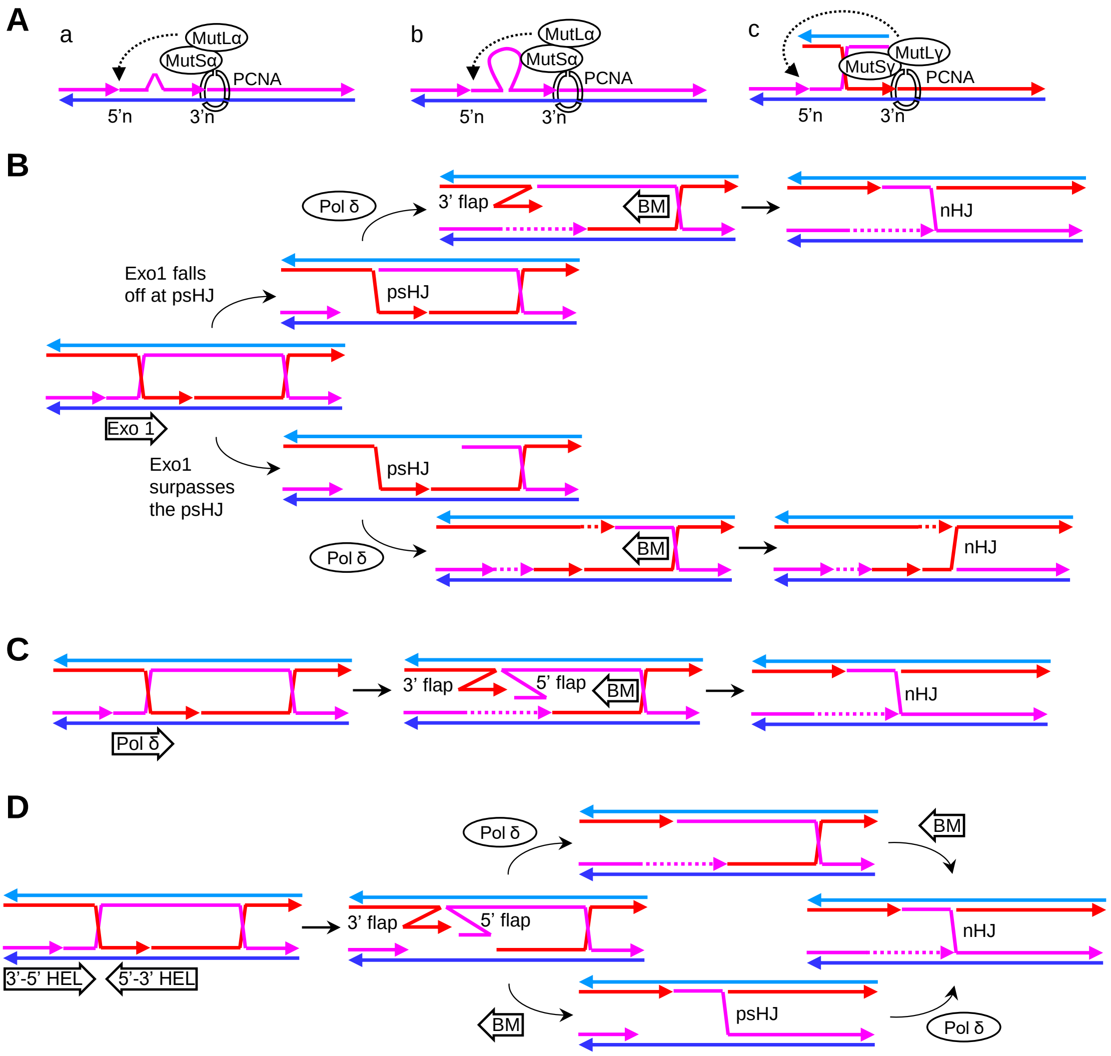

Figure S8. MMR-like mechanisms of HJ resolution by Mlh1-Mlh3 making incisions in *cis*. (**A**) Analogies between MMR and Mlh1-Mlh3 incisions in *cis*. In MMR, PCNA (and others) recognize the newly synthesized strand through unligated nicks (e.g. Okazaki fragments during lagging strand synthesis). Base-pair mismatches (a) or insertion/deletions (b; an insertion in the example) are recognized by MutS*α* (MSH2-MSH6) and MutL*α* (MLH1-PMS2 in humans; Mlh1-Pms1 in *S. cerevisiae*). When the pre-existing nick locates 3′ to the mismatch/insertion/deletion (3′n), MutL*α* makes a new incision upstream in the newly synthesized strand (5′n). MutSγ (MSH4-MSH5) and MutLγ (MLH1-MLH3) could follow an equivalent mechanism, yet with a cHJ (c) as the recognized perturbation in the dsDNA. Unlike mismatch/insertion/deletion, a cHJ in a parallel conformation predicts that the 5′n incision takes place in the other crossover strand (arched dotted arrow). (**B**) Like in MMR, the 5′→3′ exonuclease Exo1 could start degrading one strand from the 5′n. If Exo1 falls off when getting to the cHJ (upper branch), a pseudoHJ (psHJ) with one ssDNA arm is obtained. Then, Pol δ could fill in the ssDNA gap and displace the remaining crossover strand when reaching the psHJ. Once Pol δ gets to the 3′n, the psHJ is resolved, leaving a 3′-flap, which can be cut by another endonuclease. If Exo1 surpasses the cHJ, a psHJ with two ssDNA arms is obtained (lower branch). If Exo1 further degrades beyond the 3′n coordinate, flap endonucleolysis is not required and Pol δ can fill in the two ensuing ssDNA gaps. (**C**) Pol δ could also resolve the cHJ in an Exo1-independent fashion through strand displacement while nick translating from the 5′n. This results in a 5′- and a 3′-flap, which need further processing by flap endonucleases. (**D**) The concerted actions of 3′→5′ and 5′→3′ helicases could also resolve the cHJ. In this case, Pol δ would act downstream to fill in the ensuing ssDNA gap. 5′- and 3′-flap endonucleases would still be required. In the context of a dcHJ (a DPE dcHJ in the example) in which only one cHJ generated 5′n and 3′n incisions, the second cHJ can be turned into a nHJ by branch migration, provided that either of the nicks that result from gap filling (or nick translation) or flap endonucleolysis is not ligated (right half of B to D).

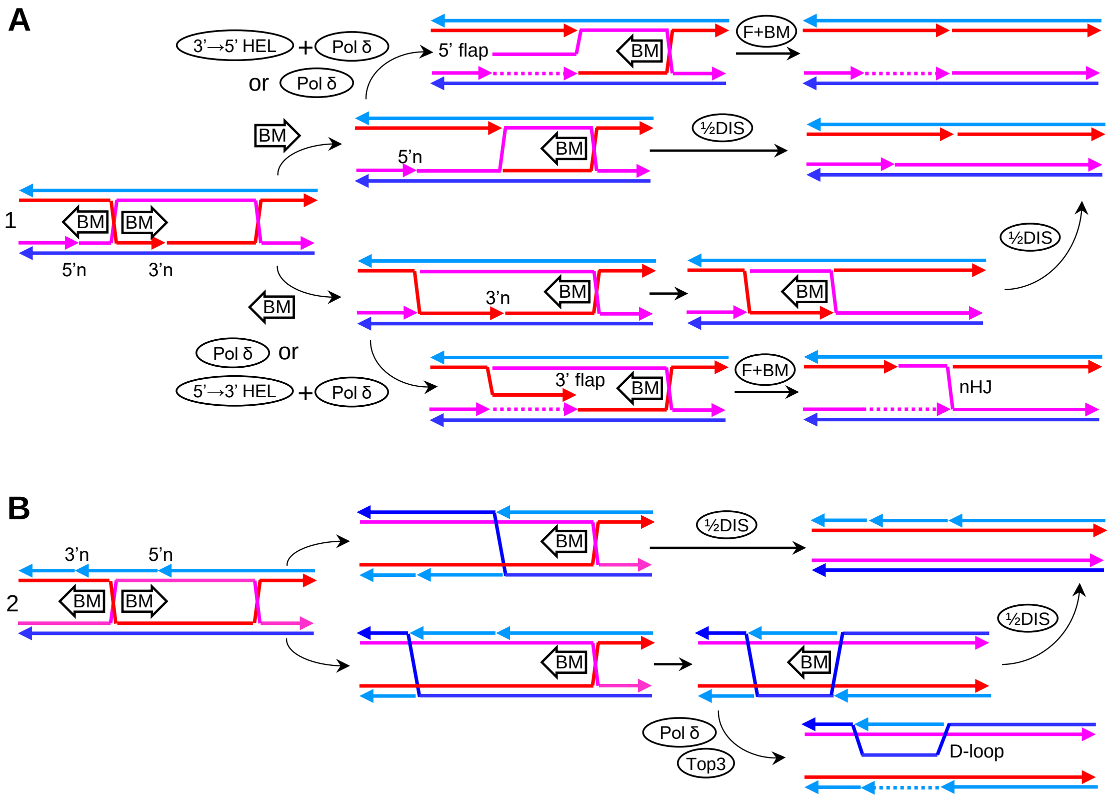

Figure S9. HJ resolution by combining branch migration and MMR-like mechanisms after Mlh1-Mlh3 makes incisions in *cis*. (**A**) Like in Figure S8, the substrate is a DPE dcHJ with Mlh1-Mlh3 making incisions in *cis* upon one crossover strands of a single HJ. After having the 5′n and 3′n nicks, branch migration comes before committing the rest of the MMR machinery. In the upper branches, migration towards the 3′n results in a nHJ that can be resolved from the 5′n by any of the MMR subpathways introduced in panels B to D of Figure S8 (top branch). In the example, the product of either 3′→5′ helicase plus Pol δ or just Pol δ is shown. The resulting 5′-flap can then be removed nucleolytically, yielding two opposite nicks that can serve as sinks for the elimination of the second HJ through branch migration (F+BM). The final genetic product would be an NCO. Alternatively, half dissolution by RTR could deal with the dncHJ without requesting the MMR machinery (middle top branch). In the lower branches, the cHJ migrates towards the 5′n. In this case, a 5′→3′ helicase would need to be recruited for strand displacement and HJ resolution (bottom branch). Pol δ could still perform this task provided that it can nick translate from a nHJ. After a nuclease deals with the 3′-flap, two non-opposing nicks are the resulting scars. This implies that branch migration does not eliminate the second cHJ but turn it into a nHJ. Branch migration without the rest of the MMR machinery would lead to a dnHJ in this scenario. (**B**) Unlike the previous substrate, Mlh1-Mlh3 has made incisions in *cis* upon one of the non-crossover strands of a single cHJ in the DPE dcHJ. In this scenario, branch migration towards either 5′n or 3′n turns the DPE dcHJ into a DPO dncHJ. Aside from half dissolution, an MMR-like mechanism could resolve the dHJ but only after co-directional branch migration turns the original DPE dcHJ into a DPE dnHJ, with one of initial non-crossover strands now shifted into the crossover strand (bottom branch). Even so, resolution would demand the cooperation between Pol δ (or a 5′→3′ helicase) and RTR (Top3 at least; see Figures 6B and S1C) and yield a D-loop in one of the NCO products.

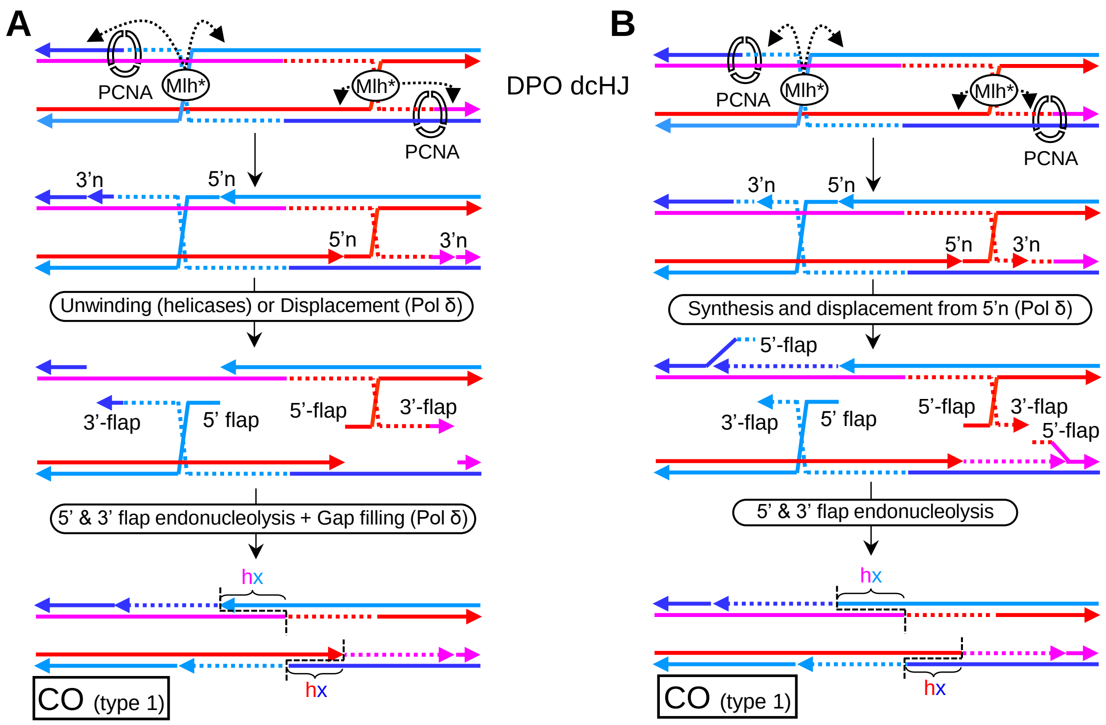

Figure S10. Alternative models for type 1 COs by double incisions in *cis* upon a DPO dcHJ. The reference model is the one shown in Figure 11A (left branch). (**A**) In this model, Mlh1-Mlh3 makes the 5'n incision downstream the newly synthesized tracts. (B) In this model, incisions occur as in Figure 11A but Pol δ synthesizes DNA beyond the 3'n. Thus, the part of the HR-driven synthesis which formed the outer hxDNA after converging branch migration is displaced as a 5'-flap to be later removed by flap endonucleases. All other steps are like in Figure 11A. See previous figure legends for abbreviations and signs.

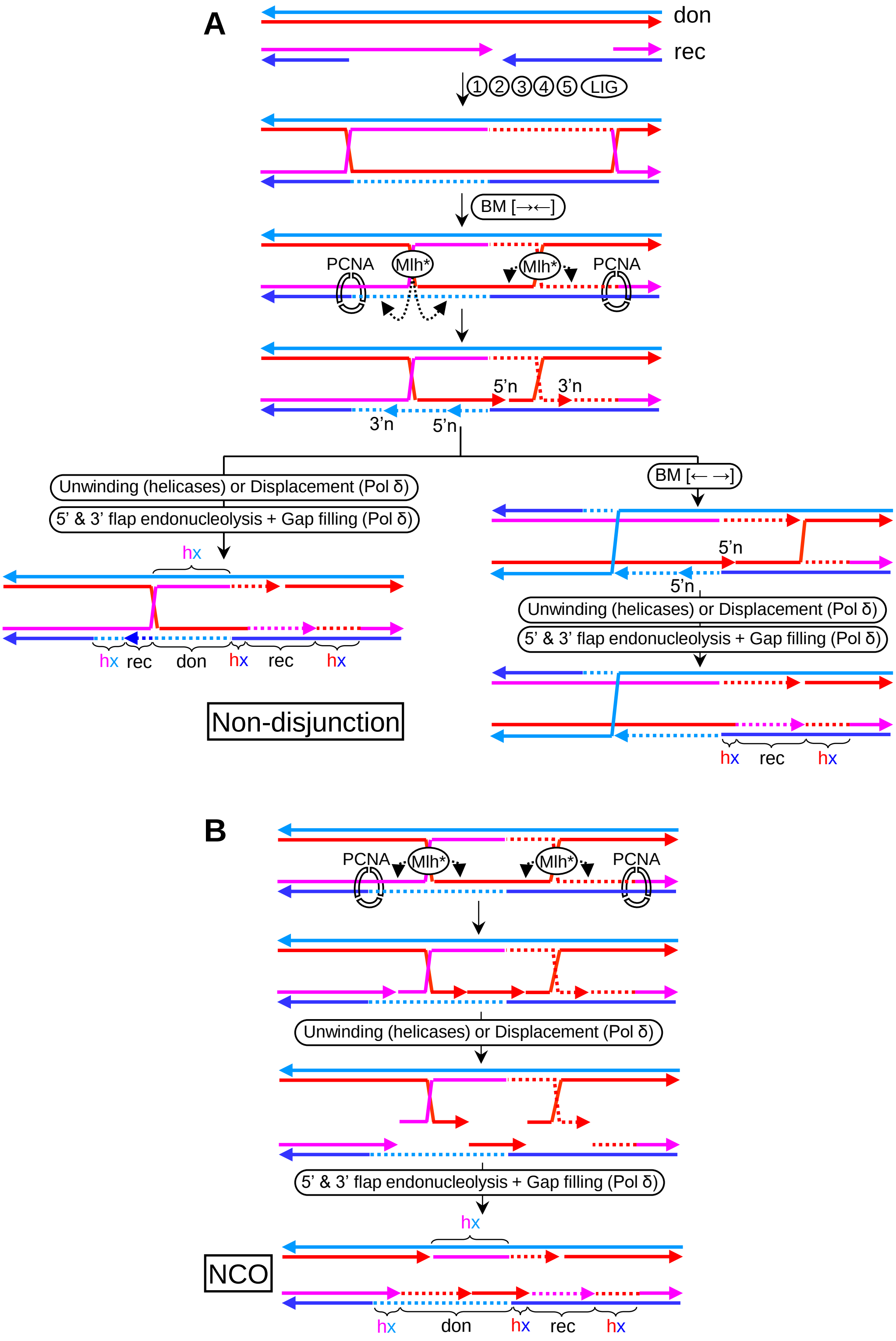

Figure S11. PCNA-conducted incisions in *cis* upon both newly synthesized strand sections struggle to resolve a DPE dcHJ. (**A**) Steps 1-5 and LIG generate a DPE dcHJ as in Figure 7A. Then, converging branch migration positions the newly synthesized tracts for PCNA-MutSγ-MutLγ activation. As a result, one HJ would carry double incisions in *cis* upon one non-crossover strand. Neither MMR-like displacement alone (left branch) nor combinations of branch migration and MMR (right branch) can easily eliminate this HJ without the aid of a canonical SSE. However, disjunction is possible if the HJ migrates to the opposite nicks on the right branch (see next figure) (**B**) Full resolution would be achieved if Mlh1-Mlh3 switched strand polarity for that HJ and incised upon the complementary strands. However, a locally complex NCO would be the expected genetic outcome. See previous figure legends for abbreviations and signs.

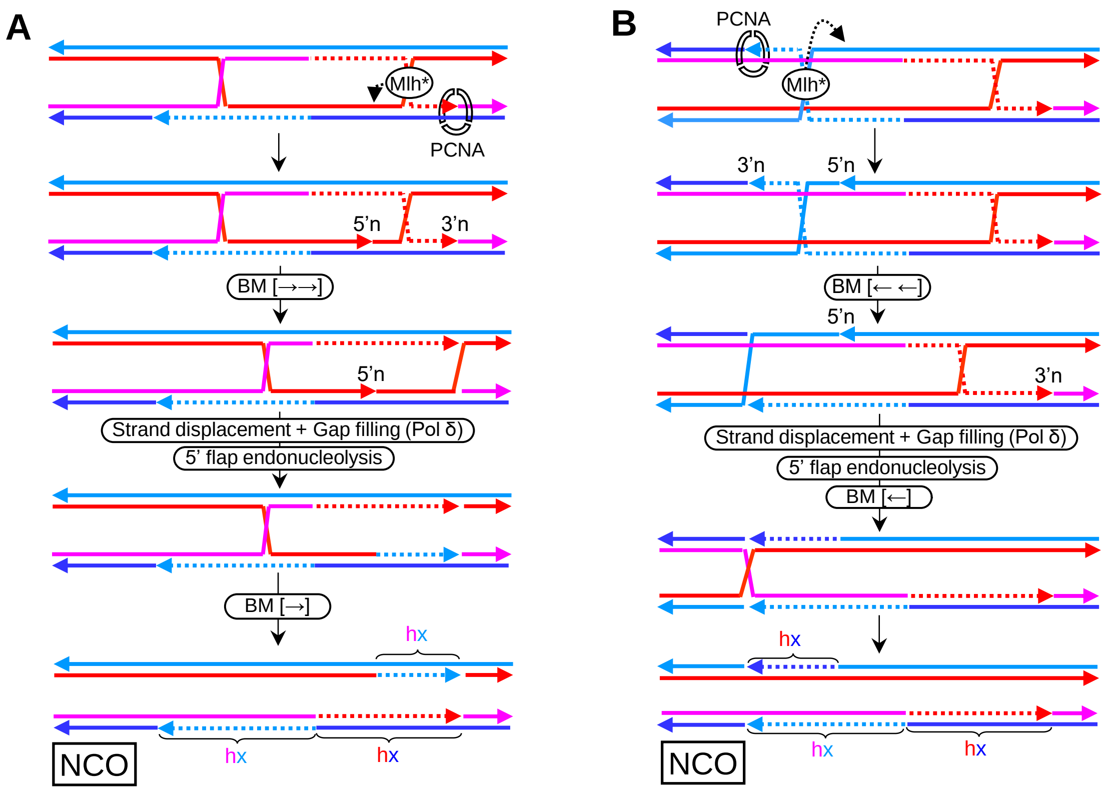

Figure S12. Incisions in *cis* in just a single cHJ could resolve DPE and DPO dcHJs into NCO products. (**A**) The substrate is a DPE dcHJ. (**B**) The substrate is a DPO dcHJ. In both cases, I consider the scenario with a pre-existing 3'n recognized by PCNA (see Figure 11A, right branch). Mlh1-Mlh3 then makes the incision 5' to the cHJ (dotted arrow). Next, the cHJ surrounded by incisions migrates towards de 3'n to become a nHJ, which is then resolved from the 5'n as in the top branch of Figure S9A. The other cHJ would be disassembled by branch migration to the ensuing opposing nicks, yielding NCOs with hx tracts. See previous figure legends for abbreviations and signs.
